# Invasive raccoon (*Procyon lotor*) and raccoon dog (*Nyctereutes procyonoides*) as potential reservoirs of tick-borne pathogens: data review from native and introduced areas

**DOI:** 10.1186/s13071-022-05245-3

**Published:** 2022-04-11

**Authors:** Izabella Myśliwy, Agnieszka Perec-Matysiak, Joanna Hildebrand

**Affiliations:** Department of Parasitology, Faculty of Biological Sciences, University of Wrocław, Wrocław, Poland

**Keywords:** Invasive species, Raccoon dog, *Nyctereutes procyonides*, Raccoon, *Procyon lotor*, Tick-borne pathogens, Vector-borne pathogens, Wildlife

## Abstract

In recent decades, populations of the raccoon (*Procyon lotor*) and the raccoon dog (*Nyctereutes procyonides*) have increased and adapted to peri-urban and urban environments in many parts of the world. Their ability to rapidly colonize new territories, high plasticity and behavioral adaptation has enabled these two species to be considered two of the most successful invasive alien species. One of the major threats arising from continually growing and expanding populations is their relevant role in maintaining and transmitting various vector-borne pathogens among wildlife, domestic animals and humans. According to the WHO, over 17% of infectious diseases are vector-borne diseases, including those transmitted by ticks. Every year tick-borne pathogens (TBPs) create new public health challenges. Some of the emerging diseases, such as Lyme borreliosis, anaplasmosis, ehrlichiosis, babesiosis and rickettsiosis, have been described in recent years as posing important threats to global health. In this review we summarize current molecular and serological data on the occurrence, diversity and prevalence of some of the TBPs, namely *Babesia*, *Theileria*, *Hepatozoon*, *Borrelia*, *Rickettsia*, *Bartonella*, *Anaplasma* and *Ehrlichia*, that have been detected in raccoons and raccoon dogs that inhabit their native habitats and introduced areas. We draw attention to the limited data currently available on these invasive carnivores as potential reservoirs of TBPs in different parts of the world. Simultaneously we indicate the need for more research in order to better understand the epidemiology of these TBPs and to assess the future risk originating from wildlife.

**Graphical Abstract:**

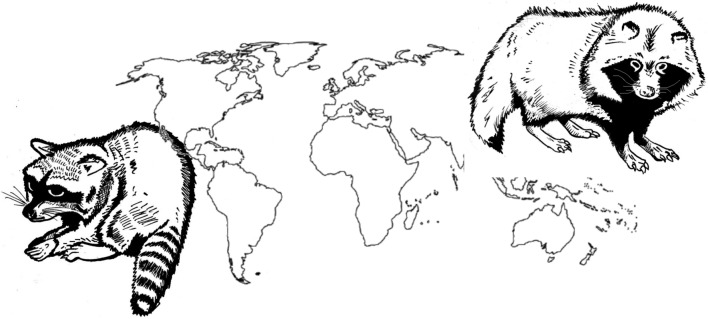

## Background

Wildlife species undisputedly serve as prime reservoirs of vector-borne pathogens. Invasive alien species in particular may play an important role in this context as they provide pathogens with opportunities to increase their abundance in the environment and spread their geographical and host range. In the future this may result in the bidirectional transmission of pathogens between wildlife and domestic animals [1–3]. This unrestricted flow of new pathogens may also have an impact on human health. In recent years, due to urbanization, climate change and the destruction of natural ecosystems, the populations of many wildlife species have increased and adapted to environments in close proximity to human populations and domestic animals [4]. Therefore, investigations on the distribution of pathogens and the dynamics of infections among wildlife and domestic animals are of great importance for a better understanding of their epidemiology [4–6].

The raccoon (*Procyon lotor*) is a North American member of the Procyonidae family and was introduced to Europe in the 1930s for fur farming and hunting, and as a pet [7, 8]. The species rapidly proliferated and spread across Europe [9–13]. In Japan, the raccoon was first introduced in the 1960s where, after the spectacular success of the animated cartoon ‘Rascal raccoon’ in 1977, it was imported from North America and popularized as a pet [10, 14]. The raccoon is highly adaptable to varying environmental conditions and is a host to numerous human pathogens, including the nematode *Baylisascaris procyonis* that is a causative agent of a severe ocular and neurological illness in many species of animals as well as in humans [12, 15, 16]. It has been confirmed that this mesocarnivore has synanthropic potential not only in its native areas but also in territories where it has been newly introduced [16–18].

The raccoon dog (*Nyctereutes procyonoides*) is a member of the Canidae family and is native to eastern Asia. There are six distinguished subspecies: *Nyctereutes procyonoides* Gray, 1834; *N. p. orestes* Thomas, 1923; *N. p. koreensis* Mori, 1922; *N. p. ussuriensis* Matschie, 1907; *N. p. viverrinus* Temminck, 1838; and *N. p. albus* Beard, 1904. This invasive carnivore was introduced into Europe for its fur in the middle of the twentieth century [19]. The ability of the raccoon dog to adapt to various environmental conditions and its high behavioral plasticity and reproductive capacity are the prime factors driving its colonizing success in Europe. They are an important reservoir of numerous zoonotic pathogens which may pose a threat to public health as well as to the biodiversity of native fauna. In addition to the red fox, in central Europe the raccoon dog can also act as a definitive host for the zoonotic parasite* Echinococcus multilocularis*, which causes alveolar echinococcosis, considered to be one of the most dangerous zoonoses [2, 20–22].

The increasing prevalence and transmission of tick-borne diseases (TBDs) are major public health issues, as over 17% of infectious diseases, including TBDs, are vector-borne. *Borrelia* spp*.*, *Anaplasma* spp., *Rickettsia* spp*.*, *Ehrlichia* spp. and *Babesia* spp. are emerging tick-borne pathogens which are highly important in terms of animal and human health worldwide [23, 24]. Raccoons and raccoon dogs have been shown to gradually spread their geographical range and colonize non-native territories and to be able to reach high population density within a short time, thereby playing a significant role in pathogen circulation. Some studies have shown that species introduced into a new environment often lose their own parasites during the course of establishing a new population (Enemy Release Hypothesis) [25] but that they also encounter and accumulate parasites which occur in the newly colonized areas. The very few publications included in the analysis presented in this review refer to both the raccoon and raccoon dog as introduced species that serve as potential reservoirs of tick-borne pathogens outside their native habitat, particularly in Europe where research has been focused principally on intestinal microparasites and helminth identification [26–31].

The aim of this review was to provide an overview of published data on raccoons and raccoon dogs as wildlife reservoirs and possible sentinels for tick-borne pathogens of bacterial and parasitic origin in their native and introduced habitats. Simultaneously, we indicate the importance of and direction for future research based on key gaps in current knowledge.

## Data sources

Publications providing data on the tick-borne pathogens reported in raccoons and raccoon dogs worldwide were identified using search engines and the Web of Science, Scopus and Google Scholar databases. The search results were manually checked and verified individually. All of the included articles were written in English and Japanese and were published between 1972 and 2021 in scientific journals. This review does not include abstracts from conferences or dissertations.

## Molecular and serological data

### Raccoon (*Procyon lotor*)

#### *Babesia* spp./*Theileria* spp.

Several *Babesia* parasites have been confirmed to potentially infect raccoons. Before molecular testing, the *Babesia* species parasitizing raccoons was named *B. lotori* based on microscopic observations [32]. In Japan, where raccoons are a non-native species, molecular studies confirmed *Babesia* sp. (from the *Babesia* sensu stricto [s.s.] group), *Babesia microti*-like and also *Babesia* species similar to *B. lotori*. The *B. microti*-like parasite was reported in two raccoons from Hokkaido, Japan, and despite the capture of 372 raccoons, only 24 were examined for the presence of this protozoan. All of the animals selected for examination had a significant splenomegaly, which is one of the clinical manifestations of babesiosis. DNA sequences extracted from two blood samples collected from raccoons testing positive for this protozoan were found to be identical to those from the USA, based on small subunit ribosomal DNA (SSU-rDNA) analysis, leading to the conclusion that this pathogen might have been introduced to Japan together with the raccoon from North America [33–35]. In the studies undertaken by Jinnai et al. [35], six out of 348 (1.7%) blood samples collected from raccoons obtained from Hokkaido gave PCR-positive signals for the presence of *Babesia* DNA. This study identified, for the first time, five unknown parasites belonging to the *Babesia* sp. from feral Japanese raccoons. Four sequences were classified into a novel group within *Babesia* genus (Clade 1) and one sequence was found to be classified into Clade 2 which also contained *Babesia* sp. found in the ixodid tick from Japan as well as *Babesia* sp., *B. divergens* and *B. odocoilei* reported in raccoons from the USA. These results indicated that new *Babesia* parasites may have established a new life-cycle in Japanese feral raccoons. Information provided by studies conducted in the USA confirmed that there are four putative piroplasm species present in raccoons from the USA (i.e. *B. lotori*, *B. microti*-like, a novel *Babesia* s.s. and a novel western *Babesia* sp.) with an additional fifth species found only in the Japanese population of raccoons [36, 37]. *Babesia microti*-like was the most common piroplasm detected in raccoons from the USA. This parasite was found for the first time in a raccoon from Massachusetts [38]. High prevalence has been reported in raccoons from Florida (82.4%) and North Carolina (84%), Minnesota and Colorado (66%). The results of studies undertaken by Garrett et al. [37] also showed high prevalence (62%) of *B. microti*-like in raccoons sampled from various locations in the USA and Canada. The survey conducted by Modarelli et al. [39] revealed for the first time the presence of the *B. microti* in raccoons from Texas (33.3%), with the reported sequence resembling one isolated from raccoons in Florida and Northern USA. Additionally, two different *Babesia* species have been detected: *Babesia* sp. Coco and another *Babesia* spo. which most closely resembles *Babesia* sp. AJB-1006 detected in a raccoon in Illinois [36, 37, 39–41]. *Babesia lotori* (previously referred to as *Babesia* s.s. and Babesia sp. AJB-2006) has been found in a single raccoon from Illinois that had clinical symptoms, and in raccoons from Minnesota and Colorado, North Carolina and various other states in the USA [36, 37, 40, 42]. No data on potential tick vectors for any *Babesia* spp. of raccoons in the USA and Japan are currently available. Only a few individuals of European raccoons in Austria and Spain have been tested for *Babesia* sp*.,* and none of these were found to be infected with this protozoan [28, 43]. The nomenclature of the *Babesia* species detected in raccoons is still inconsistent.

#### *Hepatozoon* spp.

The presence of *Hepatozoon* spp*.* in raccoons was demonstrated by molecular methods in surveys carried out in the USA [39, 44]. *Hepatozoon canis* was reported for the first time in the European population of this carnivore in Spain, with an overall prevalence of 2.6%. This study is the first and the only study of this parasite infection in raccoons from Europe [45].

#### *Borrelia* spp.

Most of the data on this spirochete in raccoons originates from the USA and is based on the results of serological testing [46–53]. Antibodies against *Borrelia burgdorferi*, *B. lonestari* or *B. turicatae* were detected. Yabsley et al. [50] attempted to confirm the seropositive results by the PCR method; however, no *Borrelia* DNA was detected during molecular testing. The molecular results from studies carried out by Tufts et al. [54] show the presence of *B. burgdorferi* only in one out of 39 raccoons. The only study on this spirochetal infection in raccoons from introduced areas was conducted in Japan, in which only one sample was seropositive for both *Borrelia afzelii* (0.1%) and *Borrelia garinii* (0.1%) [54, 55].

#### *Rickettsia* spp.

Most of the studies on the detection of *Rickettsia* in raccoons were conducted in the USA using serological methods, resulting in the detection of *Rickettsia rickettsii*, *R. montana*, *R. parkeri* and *R. bellii* 369-C strain. The most frequently detected species was *R. rickettsii*, which is an etiological agent of Rocky Mountain spotted fever (RMSP) in North and South America [51, 52, 54, 56–61]. Molecular research carried out in Japan revealed the presence of *Rickettsia japonica*, *R. tsutsugamushi*, *R. felis*, *R. heliongjiangensis/R. japonica*, *R. amblyommi*, *R. helvetica* and *Rickettsia* sp. Hj126 [55, 62, 63]; in these studies, a high number of animals were tested (*n* = 699, *n* = 752 and *n* = 194, respectively). *Rickettsia japonica* is a causative agent of RMSF in Japan. All detected species were found to be pathogenic to humans, with the exception of *Rickettsia* sp. Hj126 whose pathogenicity is unknown. European populations of raccoon have not yet been examined.

#### *Bartonella* spp.

Little is known about infection by this pathogen in raccoons. The results of molecular research in the USA demonstrated the presence of the DNA of *Bartonella rochalimae*, *B. henselae*, *B. koehlerae* and *B. berkhoffii* in samples collected from raccoons. The dominant detected species was *B. henselae*, which is a causative agent of cat-scratch disease in humans [64–67]. Researchers in Canada were the first to identify lesions associated with *Bartonella* infection in a raccoon. The species identified in this animal was closely related to *Bartonella taylorii* [68]. A study in Japan found no *Bartonella* species in 977 blood samples collected from raccoons [69]. There is no research data currently available on the occurrence of *Bartonella* among raccoons introduced into Europe.

#### *Anaplasma* spp.

Molecular and serological methods have confirmed *Anaplasma* infection among raccoons from the USA, with the results showing that raccoons may be infected with *Anaplasma phagocytophilum*. However, in these studies, the seropositive results were not always confirmed by PCR tests [50, 54, 70, 71]. In Japan, molecular studies undertaken by Sashika et al. [72] confirmed for the first time the presence of *Aanaplasma bovis* in blood from raccoons, with pathogen DNA detected in 36 out of 699 examined samples; no DNA of *A. phagocytophilum* was found during that study. These results suggest that raccoons could be a potential reservoir for *A. bovis*. Another study showed a seropositive reaction towards *A. phagocytophium* in one raccoon sample, although PCR testing did not confirm this result [73]. In Europe, a limited number of molecular studies have been conducted, on raccoons from Austria, Czech Republic, Germany and Poland [6, 28, 74]; however, *A. phagocytophilum* DNA was found only in one raccoon that originated from Poland.

#### *Ehrlichia* spp. and *Candidatus* Neoehrlichia spp.

In the USA, the most commonly used methods to detect *Ehrlichia* in raccoons have been serological methods. Seropositive results were obtained for *Ehrlichia canis* and *Ehrlichia chaffeensis* in a number of studies, but almost all results were PCR negative with the exception of one sample that was seropositive for *E. canis*. Both *E. canis* and *E. chaffeensis* are etiological agents of monocytic ehrlichiosis [50, 51, 54, 71, 75, 76]. A number of molecular studies have been carried out in Europe. Studies conducted in Austria and Spain targeted the detection of *E. canis*, which infects wild carnivores and domestic dogs worldwide [28, 45]. In the Austrian study, only four individuals were examined and no pathogen was detected. However, in the Spanish study, 194 individuals were tested and the prevalence of *E. canis* sp. DNA was 2.6%. DNA of *Ehrlichia* sp. was not detected in any of 15 raccoons examined from the Czech Republic [6] (see also [77]). Only two studies have been performed to detect *Ehrlichia* in Japanese raccoons [72, 73]. From the 187 animals examined by Inokuma et al. [73], only one and three raccoons showed a serological reaction to *E. canis* and *E. chaffeensis*, respectively, but PCR testing did not confirm these results. A molecular survey undertaken by Sashika et al. [72] showed no presence of either *E. canis* or *E. chaffeensis* DNA in 699 tested animals. *Candidatus* Neoehrlichia lotoris has been detected only in raccoons from the USA in which its prevalence is quite high—53.3% [71] and 67% [78]. It has been confirmed that this species is closely related to *Candidatus* Neoehrlichia mikurensis, and it was originally named as a novel *Ehrlichia-*like organism based on* 16S* rRNA gene sequence. As a result, the raccoon is considered to be a natural host of *Candidatus* Neoehrlichia lotoris [71, 78, 79]. Surveys from Poland, Germany and the Czech Republic did not show any presence of *Candidatus* Neoehrlichia sp. DNA in the examined samples [6, 74].

A detailed summary of currently available data on tick-borne pathogens (TPBs) in the raccoon is provided in Table 1.

**Table 1:** Tick-borne pathogens of parasitic and bacterial origin detected in raccoon (*Procyon lotor*) in its native and introduced range

TBPs	Species/genospecies	Locality	Prevalence	Diagnostic test	References
*Babesia*/*Theileria* spp.	*B. microti*-like	USA-native	1/1 (100%)	PCR	[38]
	*B. microti*-like	Japan-introduced	2/24 (8.3%)	PCR	[34]
	*Babesia* sp.	USA-native	1/1 (100%)	PCR	[42]
	*B. microti*-like	USA-native	34/41 (84%)	PCR	[40]
	*Babesia* sp.		37/41 (90%)		
	*Babesia* sp*.*	Japan-introduced	6/348 (1.7%)	PCR	[35]
	*Theileria* sp.		0/348		
	*B. microti*-like		0/348		
	*B. microti*-like	USA-native	14/17 (82.4%)	PCR	[41]
	*B. microti*-like^a^	Austria-introduced	0/4	PCR	[28]
	*B. microti*-like	USA-native	70/106 (66%)	PCR	[36]
	*Babesia* sp.		11/106 (10%)		
	*B. microti*-like	USA/Canada	490/699 (70%)	PCR	[37]
	*Babesia* sp*.*		170/699 (24%)		
	*B. microti*	USA-native	5/15(33.3%)	PCR	[39]
	*B. microti*	USA-native	0/3	PCR	[54]
	*Babesia* sp.	Spain-introduced	0/2	PCR	[43]
	*B. vulpes*		0/2		
*Hepatozoon* spp.	*Hepatozoon* sp.	USA-native	4/4 (100%)	PCR	[44]
	*H. canis*	Spain-introduced	5/194 (2.57%)	PCR	[45]
	*Hepatozoon* sp.	USA-native	3/15 (20%)	PCR	[39]
	*H. canis*	Spain-introduced	0/2	PCR	[43]
	*H. felis*		0/2		
	*H. martis*		0/2		
*Borrelia* spp.	*B. burgdorferi*	USA-native	1/21 (4.8%)	IFAT	[46]
	*B. burgdorferi*	USA-native	75/370 (20%)	ELISA	[47]
	*B. burgdorferi*	USA-native	23/87 (26%)	IFAT	[48]
	*B. burgdorferi*	USA-native	9/200 (4.5%)	IFAT	[49]
	*Borrelia* sp*.*	USA-native	IFAT 69/156 (44.23%)	IFAT/PCR	[50]
			PCR 0/169		
	*B. afzelii*	Japan-introduced	1/752 (0.1%)	IIA	[55]
	*B. garinii*		1/752 (0.1%)		
	*B. lonestari*	USA-native	1/19 (5.3%)	IFAT	[51]
	*B. burgdorferi*	USA-native	0/30	IFAT	[52]
	*B. turicatae*	USA-native	2/25 (8%)	Immunobloting	[53]
	*B. burgdorferi*	USA-native	1/39 (2.6%)	PCR	[54]
	*B. miyamotoi*		0/39		
*Rickettsia* spp.	*R. rickettsi*	USA-native	17/94 (18.1%)	CF	[56]
	*R. rickettsi*	USA-native	35/129 (27.1%)	IFAT	[57]
	*R. montana*		8/129 (6.2%)		
	*R. bellii* 369-C strain		9/129 (6.9%)		
	*R. rickettsi*	USA-native	55/120 (45.8%)	micro-IF	[58]
	*R. montana*		1/120 (0.8%)		
	*R. bellii* 369-C strain		2/120 (1.7%)		
	*R. rickettsi*	USA-native	3/14 (21.4%)	MAT	[59]
	*R. helvetica*	Japan-introduced	11/699 (1.6%)	PCR	[62]
	*R. felis*		1/699 (0.1%)		
	*R. heliongjiangensis*/*R. japonica*		1/699 (0.1%)		
	*R. typhi*	USA-native	0/9	IFAT	[60]
	*R. japonica*	Japan-introduced	14/752 (1.9%)	IIA	[55]
	*R.* tsutsugamushi		39/752 (5.2%)		
	*R. parkeri*	USA-native	14/19 (73.7%)	IFAT	[51]
	*R. amblyommi*	Japan-introduced	3/194 (1.5%)	PCR	[63]
	*Rickettsia *sp. *Hj126*		3/194 (1.5%)		
	*R. helvetica*		1/194 (0.5%)		
	*R. rickettsi*	USA-native	3/30 (10%)	IFAT	[52]
	*Rickettsia* sp.	USA-native	0/1	IFAT	[61]
	*Rickettsia* sp.	USA-native	3/39 (7.7%)	PCR	[54]
*Bartonella* spp.	*B. rochalimae*	USA-native	11/42 (26%)	PCR	[65]
	*Bartonella* sp*.*	Japan-introduced	0/977	PCR	[69]
	*B. henselae*	USA-native	12/37 (32.4%)	PCR	[66]
	*B. koehlerae*		1/37 (2.7%)		
	*B. clarridgeiae*		0/37		
	*B. rochalimae*	USA-native	11/186 (5.9%)	PCR	[67]
	*B. berkhoffii*		3/186 (1.6%)		
	*Bartonella* sp.	USA-native	0/39	PCR	[54]
	*B.taylorii-like*	Canada-native	1/1 (100%)	PCR	[68]
*Anaplasma* spp.	*A. phagocytophilum*	USA-native	IFAT 51/57 (89.5%)	IFAT/PCR	[70]
			PCR 14/57 (24.6%)		
	*A. phagocytophilum*	USA-native	IFAT 1/60 (1.7%)	IFAT/PCR	[71]
			PCR 0/60		
	*A. phagocytophilum*	Japan-introduced	IFAT 1/187 (0.5%)	IFAT/PCR	[73]
			PCR 0/9		
	*A. phagocytophilum*	USA-native	IFAT 1/156 (0.64%)	IFAT/PCR	[50]
			PCR 0/169		
	*A.phagocytophilum*	Japan-introduced	0/699	PCR	[72]
	*A. bovis*		36/699 (5.15%)		
	*Anaplasma* sp*.*	Austria-introduced	0/4	PCR	[28]
	*Anaplasma* sp*.*	Czech Republic-introduced	0/15	PCR	[6]
	*A. phagocytophilum*	Poland-introduced	1/78 (1.3%)	PCR	[74]
		Germany-introduced	0/40		
	*A. phagocytophilum*	USA-native	15/39 (38.5%)	PCR	[54]
	*A. marginale*		0/39		
*Ehrlichia* spp.	*E. chaffeensis*	USA-native	IFAT 9/43 (21%)	IFAT	[75]
	*E. chaffeensis*	USA-native	IFAT 83/411 (20%)	IFAT/PCR	[76]
			PCR 0/20		
	*E. canis*	USA-native	IFAT 13/60 (21.7%)	IFAT/ PCR	[71]
			PCR 1/60 (1.7%)		
	*E. chaffeensis*		IFAT 23/60 (38.3%)		
			PCR 0/60		
	*E. ewingii*		PCR 0/60		
	*E. canis*	Japan-introduced	IFAT 1/187 (0.5%)	IFAT/PCR	[73]
			PCR 0/9		
	*E. chaffeensis*		IFAT 3/187 (1.6%)		
			PCR 0/9		
	*E. chaffeensis*	USA-native	IFAT 49/156 (31.41%)	IFAT/PCR	[50]
			PCR 0/169		
	*E. canis*		IFAT 18/156 (11.53%)		
			PCR 0/169		
	*E. ewingii*		0/169 PCR		
	*E. chaffeensis*	USA-native	8/19 (42.1%)	IFAT	[51]
	*E. chaffeensis*	Japan-introduced	0/699	PCR	[72]
	*E. canis*		0/699		
	*E. canis*	Austria-introduced	0/4	PCR	[28]
	*Ehrlichia* sp*.*	Czech Republic-introduced	0/15	PCR	[6]
	*E. canis*	Spain-introduced	5/194 (2.57%)	PCR	[45]
	*E. canis*	USA-native	0/39	PCR	[54]
	*E. ewingii*		0/39		
	*E. chaffeensis*		0/39		
*Candidatus* Neoehrlichia sp.	*Candidatus* Neoehrlichia lotoris	USA-native	32/60 (53.3%)	PCR	[71]
	*Candidatus* Neoehrlichia lotoris	USA-native	131/197 (67%)	PCR	[78]
	*Candidatus* Neoehrlichia sp.	Czech Republic-introduced	0/15	PCR	[6]
	*Candidatus* Neoehrlichia sp.	Poland-introduced	0//78	PCR	[74]
		Germany-introduced	0/40		

Prevalence and diagnostic tests are included for each reference

CF, Complement-fixing antibodies; ELISA, enzyme-linked immunosorbent assay; IFAT, indirect fluorescent antibody test, IIA, indirect immunoperoxidase assay; MAT, microaglutination antibody test; PCR, polymerase chain reaction

^a^*B. microti-*like name was used for all sequences belonging to *B. microti* group and reported by authors as *B.* cf. *microti*

### Raccoon dog (*Nyctereutes procyonides*)

#### *Babesia* spp./*Theileria* spp.

The first molecular report of *B. microti-*like in wild raccoon dogs in South Korea indicated that these canids may play an important role as a source of piroplasm infection for both domestic dogs and humans [80]. However, in a study undertaken several years later in South Korea, Hong et al. [81] did not confirm any *B. microti-*like PCR-positive samples originating from 23 raccoon dogs. Studies on *Theileria* spp. have been conducted only in South Korea, and did not show the presence of this protozoan in the examined blood samples from raccoon dogs [82]. In Europe, the results of research conducted by Duscher et al. [28] were the first confirmation of *B. microti*-like in an introduced population of raccoon dogs.

#### *Hepatozoon* spp.

To date there have been no studies conducted on the detection of *Hepatozoon* spp. in raccoon dogs in either native or introduced areas.

#### *Borrelia* spp.

A study in South Korea using molecular techniques resulted in the first report of *B. theileri* in raccoon dogs [83]. This study also identified *Haemaphysalis flava*, a dominant species of a tick that infests raccoon dogs in South Korea. The results of this survey indicated that *B. theileri* can infect not only ungulate species but also canine species, such as raccoon dogs. Further studies are needed to define the role of this carnivore as a potential reservoir of *B. theileri* [22]. Molecular studies undertaken by Wodecka et al. [84] on European raccoon dogs in western Poland revealed that eight out of 28 tested animals were positive for *Borrelia* sp., with the dominant species being *B. garinii*, followed by less prevalent *B. afezelii* and *B. valaisiana*. This study indicated that the role of raccoon dogs as a potential reservoir for the bird-adapted *B. garnii* should be thoroughly investigated. Additionally, in this same study, *Borrelia* species were identified in 20.1% of ixodid ticks collected from the raccoon dogs examined [84].

#### *Rickettsia* spp.

Studies related to *Rickettsia* species have been conducted only in the native habitat of raccoon dogs, namely Japan and South Korea. Neagari et al. [85] screened samples from 30 raccoon dogs using serological tests with the aim to detect *R. japonica* and *R. tsutsugamushi* antibodies; however, none of the examined carnivores were infected with these bacterial species. Other research carried out in South Korea identified seropositive raccoon dogs, with spotted fever group rickettsia (*R. japonica*) and typhus group rickettsia (*R. typhi*) antibodies detected in 30.5% and 41.6% of animals, respectively [86]. These results are of great importance as the YH strain antigen (*R. japonica*) used in the test on raccoon dogs is the same strain used in the detection of seropositive humans in South Korea. This study was the first time in South Korea that wild animals were used as rickettsial infection indicators [86]. Molecular studies undertaken by Han et al. [81] did not show the presence of rickettsia species in any of 15 blood samples from raccoon dogs in South Korea.

#### *Bartonella* spp.

Research on this Gram-negative bacterium has been performed only in Japan and South Korea. Early studies on *Bartonella* in Japan confirmed DNA infection in 11 out of 171 raccoon dogs; however, this pathogen was not isolated from carnivores. The amplicons obtained were most closely related to those of *B. rochalimae* which is an emerging zoonotic pathogen in Europe, South America and the USA [69, 87]. Molecular surveys of 619 Japanese raccoon dogs (*Nycetereutes procyonides viverrinus*) revealed the presence of *B. rochalimae* DNA in the blood samples examined. However, this species has never been detected in any other carnivore co-inhabiting the area with the raccoon dogs, which suggests that raccoon dogs specifically may be able to harbor this bacterium species in their blood. Nevertheless, more research is needed to confirm this hypothesis [88]. In another study, *B. henselae* DNA was detected in blood and spleen samples of raccoon dogs in South Korea [22].

#### *Anaplasma* spp.

Only two studies have been conducted in Asia on *Anaplasma* spp., both in South Korea. Han et al. [81] confirmed the first infection of *A. bovis* in Korean raccoon dogs and suggested that they may act as a natural reservoir of this pathogen. However, only 15 samples of raccoon dogs were tested in this study, and only one sample was PCR-positive for *A. bovis*. In a larger study which was carried out subsequent to that Han et al. [81], Kang et al. [22] examined 193 splenic tissue and blood samples of Korean raccoon dogs; screening by PCR showed the presence of *A. bovis* in 2.1% of the samples tested and, for the first time, the presence of *A. phagocytophilum* in 1% of samples. Studies on this bacterium have also been conducted in Europe. *Anaplasma phagocytophilum* has been confirmed in raccoon dogs from Germany [89] and Poland [90]. The study in Poland was the first in Europe that involved a large number of raccoon dogs. Testing of 68 spleen samples showed that 24 samples (35.3%) were positive for *A. phagocytophilum*. Other studies carried out in Poland did not show the presence of *Anaplasma* species [74] and neither did surveys carried out in the Czech Republic [6] and Austria [28].

#### *Ehrlichia* spp. and *Candidatus* Neoehrlichia

To date, only one study has been conducted to detect *Ehrlichia* spp. in the Korean native habitat of raccoon dogs, and none of 15 blood samples examined was positive for this pathogen [82]. However, only a small number of carnivores were examined. Studies performed on raccoon dogs in Austria [28] and Czech Republic [6] also did not show the presence of *Ehrlichia* or *Candidatus* Neoehrlichia spp. DNA. Research undertaken by Hildebrand et al. [74] revealed for the first time the presence of *Candidatus* Neoehrlichia spp. (FU98) in raccoon dogs from Poland and established the raccoon dog as a new host for this pathogen. A detailed summary of currently available data on TPBs in free-ranging raccon dogs is provided in Table 2.

**Table 2:** Tick-borne pathogens of parasitic and bacterial origin detected in free-ranging raccoon dogs (*Nyctereutes procyonoides*) in their native and introduced range

TBPs	Species/genospecies	Locality	Prevalence	Diagnostic test	References
*Babesia*/*Theileria* spp.	*B. microti*-like	South Korea-native	3/14 (21.4%)	PCR	[80]
	*B. microti*	South Korea-native	0/23	PCR	[81]
	*B*. *microti*-like^a^	Austria-introduced	5/8 (62.5%)	PCR	[28]
	*Theileria* sp.	South Korea-native	0/15	PCR	[82]
*Borrelia* spp.	*B. afzelii*	Poland-introduced	2/28 (25%)	PCR	[84]
	*B. garinii*		5/28 (62.5%)		
	*B. valaisiana*		1/28 (12.5%)		
	*B. theileri*	South Korea-native	1/142 (0.7%)	PCR	[22]
*Rickettsia* spp.	*R. japonica*	Japan-native	0/30	IFAT	[85]
	*R. tsutsugamushi*		0/30		
	*R. japonica*	South Korea-native	11/36 (30.5%)	IFAT	[86]
	*R. typhi*		15/36 (41.6%)		
	*Rickettsia* sp*.*	South Korea-native	0/ 15	PCR	[82]
*Bartonella* spp.	*Bartonella* sp.	Japan-native	0/171	PCR	[69]
	*B. henselae*	South Korea-native	2/142 (1.5%)	PCR	[22]
	*B. rochalimae*	Japan-native	44/619 (7.1%)	PCR	[88]
*Anaplasma* spp*.*	*A. phagocytophilum*	Germany-introduced	3/13 (23.2%)	PCR	[89]
	*Anaplasma* sp.	Austria-introduced	0/8	PCR	[28]
	*Anaplasma* sp.	Czech Republic-introduced	0/7	PCR	[6]
	*A. bovis*	South Korea-native	1/15 (6.6%)	PCR	[82]
	*A. phagocytophilum*	Poland-introduced	0/10 (30%)	PCR	[74]
	*A. phagocytophilum* *A. bovis*	South Korea-native	2/193 (1%) 4/193 (2.1%)	PCR	[22]
	*A. phagocytophilum*	Poland-introduced	24/68 (35.3%)	PCR	[90]
*Ehrlichia* spp.	*Ehrlichia* sp.	South Korea-native	0/15	PCR	[82]
	*E. canis*	Austria-introduced	0/8	PCR	[28]
	*Ehrlichia* sp*.*	Czech Republic-introduced	0/7	PCR	[6]
*Candidatus* Neoehrlichia spp.	*Candidatus* Neoehrlichia sp.	Austria-introduced	0/8	PCR	[28]
	*Candidatus* Neoehrlichia sp.	Czech Republic-introduced	0/7	PCR	[6]
	*Candidatus* Neoehrlichia sp. (FU98)	Poland-introduced	3/10 (30%)	PCR	[74]

Prevalence and diagnostic test are included for each reference

IFAT, indirect fluorescent antibody test, PCR, polymerase chain reaction

^a^*B. microti-*like name was used for all sequences belonging to *B. microti* group and reported by authors as *B.* cf. *microti*

## Conclusions

A summary of the data originating from research carried out mostly in the last two decades allows us to conclude that the raccoon and raccoon dog are indeed species with the potential to be competent reservoirs of numerous TBPs. However, many epidemiological aspects are still poorly understood, and more research is required. It is exceptionally noteworthy that very few studies on the incidence of TBPs in these carnivores have been conducted in introduced areas. Both animals are alien species that have been introduced into Europe, yet little or even no knowledge on the specific TBPs they may harbor is available. Therefore, many opportunities for further research still exist. Future studies should prioritize the testing of larger populations of introduced raccoons and raccoon dogs for the presence of TBPs in areas where those animals have not yet been sampled (or for which data are insufficient). Results could then be compared with those obtained from their native habitats. Moreover, the sympatric occurrence of invasive and native carnivores facilitate the inter-species transmission of pathogens and may also play a relevant role in the circulation of pathogens transmitted by ticks. Evaluation of possible cross-species transmissions, vector establishment and an insight into possible zoonotic implications appear to be essential for a better understanding of the epidemiology of TBDs and to assess the potential risk originating from these two invasive species.

## Data Availability

All data analyzed during this study is included in this published article.

## Abbreviations

TBDs: Tick-borne diseases
TBPs: Tick-borne pathogens
